# Blockchain-enabled digital twin systems in healthcare: a systematic scoping review of architectural integration, privacy, governance, ethical challenges, and regulatory solutions under GDPR and HIPAA

**DOI:** 10.3389/fdgth.2026.1898383

**Published:** 2026-08-12

**Authors:** Muhammad Farooq Shaikh, Syed Hamza Hassan, Jawwad Shamsi, Alessia Maccaro, Davide Piaggio

**Affiliations:** 1 School of Engineering, University of Warwick, Coventry, United Kingdom; 2 Department of Computer Science, National University of Computer and Emerging Sciences, Islamabad, Pakistan; 3 Department of Social Sciences, University of Naples Federico II, Napoli, Italy

**Keywords:** blockchain, clinical decision support, data integrity, digital twin, ethics, federated learning, healthcare, healthcare architecture

## Abstract

**Background and objective:**

The integration of blockchain and digital twin (DT) technologies is increasingly recognised as a promising approach for improving healthcare data integrity, interoperability, privacy, and clinical decision support. While digital twins enable dynamic patient modelling and predictive healthcare applications, blockchain provides secure data governance through decentralised trust, auditability, and access control. However, existing research remains fragmented, with limited synthesis of the architectural integration, regulatory readiness, ethical governance, and interoperability of blockchain-enabled healthcare digital twin systems. This systematic scoping review addresses these gaps by providing a comprehensive architectural and compliance-oriented analysis of the current evidence.

**Methods:**

A systematic scoping review was conducted following PRISMA 2020 guidelines using Scopus, PubMed, and Web of Science. From 148 identified records, 55 eligible studies published between 2020 and 2025 were included after duplicate removal and eligibility screening. Data were extracted on digital twin functionality, blockchain architecture, healthcare application domains, consensus mechanisms, privacy-preserving strategies, and regulatory and ethical alignment. Structured Python-based visual mapping and comparative analyses were performed to identify architectural, governance, and compliance patterns across the literature.

**Results:**

The findings demonstrate that blockchain is predominantly employed to provide access control, audit logging, data integrity, consent management, and secure data provenance within healthcare digital twin ecosystems. Patient-level and EHR-centred digital twins represented the most mature application areas, whereas cross-domain and infrastructure-level frameworks dominated early architectural exploration. The review identifies recurring compliance-oriented architectural patterns while revealing substantial gaps in clinically validated deployments, interoperability with established healthcare standards, decentralised governance models, and formal implementation of GDPR- and HIPAA-compliant engineering practices. Comparative heatmap analyses further highlight the uneven maturity of ethical governance and regulatory integration across blockchain functionalities.

**Conclusion:**

This review provides the first comprehensive compliance-oriented architectural synthesis of blockchain-enabled healthcare digital twin systems by integrating technical architecture, regulatory readiness, ethical governance, and privacy-preserving design patterns within a unified analytical framework. The proposed architectural mapping identifies critical research gaps in interoperability, governance engineering, consensus optimisation, and real-world clinical validation, providing a foundation for the development of trustworthy, GDPR/HIPAA-aligned, FHIR-compatible, and clinically interoperable healthcare digital twin ecosystems.

## 1 Introduction

The growth of digitalisation of healthcare systems has increased, both blockchain and digital twin (DT) technologies have been rapidly evolving using advanced computational paradigms. Digital twins, which are dynamic and data-driven virtual descriptions of physical objects, have become the future modelling tools in predictive modelling, personalised medicine, and optimisation of real-time systems (1–3). Furthermore, in the healthcare sector, DTs allow sustained integration of biological, behavioural, and clinical data streams, which allow forecasting the disease, simulating treatment, and monitoring it over time (3–9).

Simultaneously, blockchain technology has been adopted as a decentralised and immutable infrastructure of secure information sharing in distributed trust environment (10). Furthermore, blockchain networks provide networks with verifiable auditability, resilience to single-point failures using cryptographic primitives, consensus mechanism, and immutable ledgers due to their applicability to healthcare environments characterised by fragmented data ecosystems and multi-stakeholder governance structures (11–13).

Moreover, combination of blockchain and digital twin technology together has been proposed as a strategic foundation of reliable, interoperable, and intelligent healthcare systems (1, 4, 6, 11, 14). Additionally, recent research explains the implementation of proof of concepts in areas such as hospital management, emergency response systems, telemedicine, personalised disease prediction, and smart clinical trial platforms (3–6). Furthermore, these systems were designed to ensure secure data provenance, access control, and traceable model updates and to allow predictive analytics, dynamically virtualised patients or clinical processes (1, 14–17).

Although significant technical advances have been achieved, blockchain-based DT systems still have severe shortcomings in terms of regulation, ethics, and governance (11, 17–19). Healthcare has strict legal requirements, including the General Data Protection Regulation (GDPR) in the European Union and Health Insurance Portability and Accountability Act (HIPAA) in the US (11, 17, 20). The immutability of blockchain has possible contradictions with the GDPR requirements such as data deletion and modification rights under GDPR (17, 20, 21). Similarly, the transfer of data across border, the enforceability of smart contracts, the pseudonymisation standards and allocation of accountability in distributed networks remain to be compliance challenges that have not been resolved (17, 20, 21).

Furthermore, recent literature has concentrated predominantly on technical architectures and application scenarios instead of organised analysis of regulatory alignment, privacy-by-design and regulatory readiness to clinical implementation. In addition, the choice of consensus mechanism, off-chain storage approaches, cryptographic methodological privacy mechanisms, as well as auditability models are rarely examined in terms of engineering legal compliance.

Although several review studies have examined blockchain use in healthcare or digital twin technologies separately, no systematic synthesis of where blockchain enabled digital twins meets regulatory compliance, ethical governance, and privacy preserving-architectures has been reported. Therefore, a systematic literature review that specifically addresses the topic of regulatory readiness and privacy-by-design integration in blockchain-based healthcare digital twins is required to consolidate existing evidence and pinpoint architectural and governance gaps in the whole domain.

Finally, to mitigate this gap, the current systematic review summarises the existing literature on blockchain-based digital twin systems in healthcare with ethical governance, privacy protection frameworks, and regulatory adherence to GDPR and HIPAA. Moreover, this review will take the discussion out of the provisional innovation stage of proof-of-concept to the regulatory-grade implementation readiness through mapping architectural patterns, privacy preserving techniques and compliance strategies.

## 2 Methods

### 2.1 Study design and reporting framework

This review was carried out according to the PRISMA 2020 guidelines (22). The review protocol was predefined prior to the study, and the PRISMA checklist is provided in Supplementary File 1. The complete electronic search strategy is presented in Supplementary File 2.

### 2.2 Eligibility criteria

A systematic structure was used to define the eligibility criteria to achieve a methodological consistency with the regulatory relevance. The studies with both blockchain and digital twin technologies in the context of healthcare were included particularly related to data governance, privacy protection, auditing, or regulatory opportunities.

The full inclusion and exclusion criteria are presented in Table 1 below.

**Table 1:** Inclusion and exclusion criteria applied in study selection for the systematic review of blockchain-enabled digital twin systems in healthcare, with emphasis on regulatory compliance, privacy governance, and ethical frameworks under GDPR and HIPAA.

Criterion	Inclusion criteria	Exclusion criteria
Study design	Empirical studies (quantitative, qualitative, mixed methods), design science research with implementation evaluation, pilot clinical deployments	Editorials, commentaries, letters, conference abstracts without full text, narrative reviews
Technology scope	Studies integrating both blockchain and digital twin technologies as core components of the system architecture	Studies using only blockchain or only digital twins without integration
Healthcare context	Clinical, hospital, telemedicine, IoMT, emergency medicine, personalised medicine, healthcare governance, or health data management settings	Non-healthcare domains (e.g., manufacturing, logistics, smart cities)
Regulatory/governance relevance	Studies addressing privacy, security, auditability, data governance, ethical implications, GDPR, HIPAA, or compliance mechanisms	Purely technical performance optimisation without privacy or governance discussion
Publication type	Peer-reviewed journal articles and full conference papers	Theses, book chapters, grey literature (unless explicitly justified)
Time frame	January 2020 – December 2025	Publications prior to January 2020 and later than December 2025
Language	English	Non-English publications
Full text availability	Full-text accessible	Unavailable full text

### 2.3 Information sources and search strategy

A systematic search was conducted on three databases: Scopus, PubMed and Web of Science to have a wide coverage of biomedical, interdisciplinary, and engineering-based research in healthcare. Only studies in English language and those publications that were published not earlier than January 2020 and not later than December 2025 were included, which was also in line with the study eligibility criteria.

Main keywords included terms such as: (i) blockchain technologies (e.g., blockchain, distributed ledger, smart contracts), (ii) digital twins (e.g., digital twin, virtual patient), (iii) healthcare setting (e.g., clinical, hospital, medical), and (iv) governance and regulated constraints were used to create database-specific Boolean search strings. Each database had its search syntax modified to suit the difference in the field tags and indexing structure.

The exact Boolean strings used in the entire search strings of all databases are presented in Supplementary File 2.

### 2.4 Study selection

The retrieved articles were added to a screening database based on Excel to deduplicate them and select the studies. The process of screening was done by removing duplicate records before a two-step selection process:

- Title and abstract screening to eliminate records that failed to meet the criteria of healthcare scope, technology integration scope and publication type.
- Full-text evaluation to establish inclusion eligibility, exclusion documented together with the cause.

**Figure 1:**
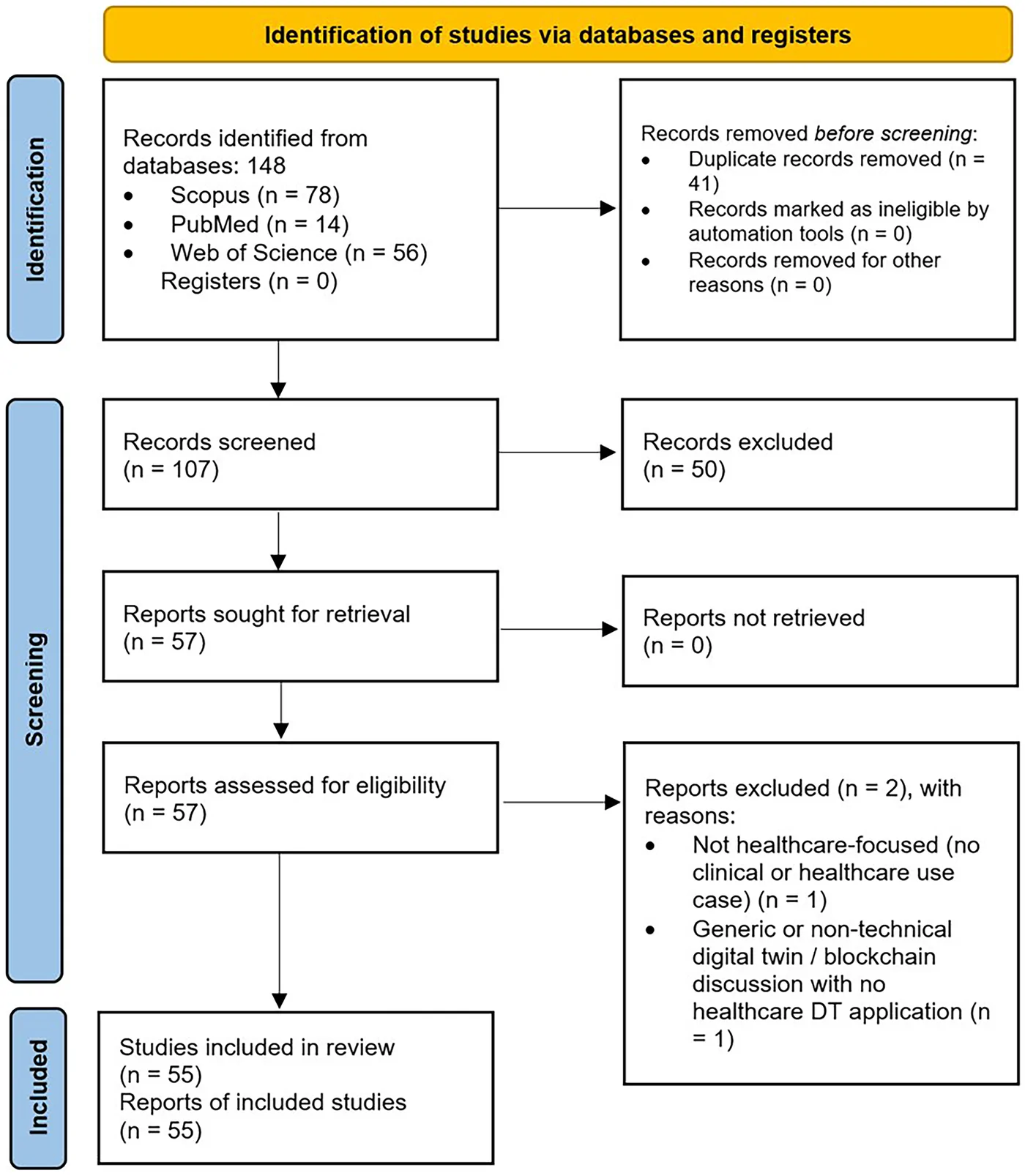
PRISMA 2020 flow diagram summarising study identification, screening, eligibility assessment, and final inclusion for the systematic review.

Predefined inclusion and exclusion criteria were applied to select the studies (Table 1). To report the screening process and final inclusion results, a PRISMA flow diagram was generated (see Figure 1 below).

### 2.5 Data extraction

An organised data extraction framework was created to provide systematic synthesis of architectural, governance, and regulatory dimensions amongst the included studies. The extraction process is mainly based on the descriptive mapping that helps in conducting comparative analysis of compliance readiness and privacy-preserving design patterns in blockchain-based digital twin (DT) healthcare systems.

Moreover, data was extracted by two reviewers who worked independently and utilised a predefined template. The template was revised several times because of pilot extraction of a subset of eligible studies to make it clear and internally consistent and to ensure that it is aligned with the review objectives. Conflicts were settled by consensus and discussion.

Furthermore, there were three main domains that were extracted. To begin with, the general study features, such as the year of publication, country in which the study was conducted, healthcare setting, and methodological design, were recorded. Secondly, the elements of the technical architecture were identified, including blockchain type (public, and private), consensus mechanism, on-chain vs. off-chain data solutions, and the extent of the digital twin implementation (patient-level, system-level, or device-level). Thirdly, and the key to this review are the governance and regulatory aspects which were combined, such as identity management models, access control mechanisms, auditability structures, privacy-preserving cryptographic techniques, and specific mentioning of GDPR, HIPAA, or other data protection standards.

Finally, the deployment phase of each system (conceptual framework, prototype, pilot implementation or real-world implementation) is documented, as well as any limitations (especially related to scalability, interoperability, and compliance with regulations) is also reported.

### 2.6 Methodological quality assessment

The quality of included studies in terms of methodological quality was measured with the help of the Mixed Methods Appraisal Tool (MMAT), version 2018 (23). The MMAT was chosen because of the diversity of the research designs included as they represented quantitative, mixed-method and design science designs (23).

All the studies were categorised based on the methodological type of the study and the five MMAT criteria were used. No numerical score was added together in accordance with the MMAT instructions. However, synthesis on appraisal findings was done descriptively. The MMAT based quality assessment is presented in Supplementary File 3.

## 3 Results

### 3.1 Study selection

A total of 148 records were retrieved using Scopus, Web of Science and PubMed via systematic search. After the elimination of duplication (*n* = 41), 107 records were screened by title and abstract. At the time of screening, 50 studies were eliminated as most of the articles were missing the key inclusion points such as: did not mention healthcare settings, no implementation or discussion of digital twin and blockchain technologies within the healthcare systems. A total of fifty-seven full-text articles were evaluated in terms of eligibility. At this point two papers were eliminated as neither had a healthcare use case, it was simply concept discussion without technical implementation (20, 24). Finally, 55 studies were included in the review. Figure 1 defines the breakdown of methodology used in the study selection.

### 3.2 Study characteristics

The total of 55 studies, which were published between 2020 and 2025, were included as evidence of the rapid emergence of blockchain-based digital twin (DT) systems in healthcare. Most of them were journal articles, and they were supplemented with conference proceedings, surveys, and reviews. The effect of this distribution is that the field process has been shifted towards more structured implementations of systems.

Moreover, the application areas of healthcare applications were broad, and digital twins were extensively used in patient-level monitoring, diagnosis, predictive modelling, and clinical decision-making across various fields in health care (1, 4, 25, 26). However, few of those articles were related to EHR/EMR integration and secure health data exchange (27–29) and remote sensing and wearable-integrated systems with IoMT-based monitoring architectures (17, 30, 31). Furthermore. healthcare digital twins that were enabled using metaverse and XR were placed in a separate category, where governance, trust, and immersive clinical simulation are commonly featured (32–35).

Additionally, literature on blockchain architectures were mainly permission-based or private models, targeting to offer secure access control, data integrity, tamper resistance, and trusted inter-institutional data exchange (4, 5, 27). In research context, blockchain was most used as an identity verification trust layer, audit logs, and consent management (6, 21, 25). However, smart contracts were traditionally proposed to implement the rules of authorisation and data-sharing (2, 18), but NFT-based solutions were considered in situations that deal with data origin tracking and ownership modelling (15, 36). Moreover, Table 2 below gives a systematic analysis of these attributes. Instead of a descriptive listing of studies, Table 2 is based on the category of studies by domain of healthcare, scope of digital twins, blockchain architecture, and regulatory orientation as the analytical framework of the further cross-domain heatmap studies.

**Table 2:** Characteristics of included studies on blockchain-enabled digital twin systems in healthcare (*n* = 55).

Study (First author, year)	Study type	Healthcare domain	Digital twin scope	Blockchain architecture	Regulatory/ethical focus
El Azzouzi et al, 2021 (27)	Conference	Clinical identity security	Patient-level DT	Immutable ledger (ID verification)	Privacy, security
Akash et al, 2022 (1)	Journal	EHR-centred healthcare	Patient-level DT	Blockchain access & integrity	GDPR, HIPAA
Arastouei et al, 2025 (37)	Journal	Healthcare data streams	Infrastructure DT	Secure DT data storage	Privacy, security
Kumari et al, 2023 (12)	Journal	Patient medical data	Cloud-based DT	Blockchain auditing	Privacy-preserving
Thakur et al, 2023 (14)	Journal	DT data streams	Environmental/device DT	Authentication & integrity	Privacy-aware
Wang et al, 2022 (38)	Perspective	Medical imaging	Imaging DT	Secure data sharing	Privacy regulation
Yi et al, 2023 (25)	Journal	EMR systems	Patient EMR DT	Verification & audit	Privacy core
Hemdan et al, 2025 (39)	Review	Healthcare DT + FL	Patient & system DT	Integrity & governance	Privacy-preserving
Vasiliu-Feltes et al, 2023 (13)	Journal	Precision health	Conceptual patient DT	Governance framework	Ethics & trust
Awan et al, 2024 (26)	Journal	Clinical patient data	Patient DT	Cryptographic access	Security & privacy
Patel et al, 2024 (40)	Survey	Cross-domain healthcare	Generic DT	Blockchain-assisted security	Privacy discussed
Mozumder et al, 2023 (32)	Journal	Metaverse healthcare	Conceptual DT	Blockchain trust layer	Ethics discussed
Zanitti et al, 2023 (9)	Conference	Oncology	Clinical DT	Privacy-preserving blockchain	GDPR-aligned
Wang et al, 2022 (41)	Journal	Mobile health	Patient DT	Traceability framework	Privacy-preserving
Qu et al, 2023 (36)	Conference	Safety monitoring	Behavioural DT	NFT-based sharing	Privacy considered
Ali et al, 2023 (33)	Journal	Metaverse healthcare	Avatar-based DT	Trust & access control	Regulatory awareness
Qayyum et al, 2023 (42)	Conference	AI-XR surgical planning	Patient-specific DT	Secure data integrity	Strong ethics focus
Bhatia et al, 2023 (2)	Conference	Metaverse DT support	Surgical planning DT	Smart contracts	Privacy & security
Amofa et al, 2024 (6)	Journal	EHR, PHR	Patient DT	Smart contracts & encryption	HIPAA-aware
Kerrison et al, 2023 (30)	Journal	IoT-enabled DT	Cloud-based DT	Identity & integrity	Privacy engineering
Samanta et al, 2022 (43)	Conference	Smart-city DT	Urban healthcare DT	Security & integrity	Security discussed
Gebreab et al, 2022 (15)	Journal	Medical device lifecycle	Device DT	NFT provenance tracking	Compliance aware
Okegbile et al, 2022 (44)	Conference	Human DT connectivity	Personalized DT	Secure communication	Privacy-centric
Pal et al, 2023 (45)	Conference	Metaverse DT security	Healthcare DT core	Integrity & access	Trust-focused
Tang et al, 2025 (5)	Journal	Secure data sharing	Prediction DT	Tamper-resistant blockchain	Secure sharing
Hulsen et al, 2024 (46)	Journal	Metaverse medicine	Hospital DT	Secure medical records	Ethics & regulation
Taleb et al, 2025 (47)	Review	AI-6G healthcare	Patient simulation DT	Governance layer	Ethics & governance
Upadrista et al, 2025 (4)	Journal	Stroke prediction	Patient DT	Consortium blockchain	GDPR-aware
Martina et al, 2024 (7)	Conference	Industry 4.0 healthcare	Hospital operations DT	Secure access exchange	Privacy & interoperability
Ali et al, 2022 (48)	Journal	Web3 smart healthcare	System-level DT	Secure coordination	Privacy-preserving
Bamakan & Banaeian Far et al, 2025 (34)	Journal	Distributed DT	Healthcare services DT	NFTs & DAOs	Privacy-focused
Bhattacharya et al, 2025 (49)	Conference	Healthcare education	Patient DT twins	Permissioned blockchain	Privacy-controlled
Chaddad et al, 2025 (50)	Journal	Medical education	Training DT	Secure record access	Regulatory compliance
Erol et al, 2025 (21)	Journal	Precision dentistry	AI-driven DT	Consent & audit trails	GDPR/HIPAA
Gao et al, 2025 (28)	Journal	Secure EHR sharing	EHR DT	Blockchain encryption	Strong privacy
Ghayvat et al, 2023 (51)	Journal	IoT ecosystem	Smart hospital DT	Blockchain-based control	Privacy-centric
Gomaa et al, 2024 (29)	Conference	IoT-EHR integration	Virtual patient IDs	Blockchain integrity	Privacy-focused
Jain et al, 2024 (31)	Journal	IoMT security	Patient DT	Immutable ledger	Security & trust
Sharma et al, 2025 (52)	Journal	Cross-sector DT	System DT	Secure cloud storage	Strong privacy
Mohanraj et al, 2025 (53)	Review	Cross-sector DT	Healthcare system DT	Data lifecycle management	Regulatory challenges
Naeem et al, 2025 (54)	Conference	AI-metaverse healthcare	Smart-city DT	Secure access & sharing	Ethical governance
Pişirgen et al, 2025 (55)	Conference	Precision IoT DT	Patient precision DT	Distributed ledger	Data privacy
Repetto et al, 2025 (3)	Journal	Predictive healthcare	AI-assisted DT	Knowledge-sharing blockchain	Compliance-focused
Ruiu et al, 2024 (18)	Journal	Biometric DT	Human digital twin	NFT-secured biometrics	Privacy & identity
Sanchez et al, 2025 (56)	Journal	Biobank DT	NFT-based data control	Ownership & consent	Regulatory-aligned
Saratkar et al, 2025 (19)	Review	Personalized medicine	Patient genomic DT	Secure sharing	Ethics & interoperability
Selvagayathri et al, 2025 (57)	Conference	Quantum secure DT	Remote monitoring DT	Quantum blockchain	Privacy-focused
Shankhdhar et al, 2025 (58)	Journal	Personalized security	Personalized DT	Secure blockchain access	GDPR/HIPAA
Joo et al, 2024 (59)	Journal	Federated healthcare DT	Imaging DT	Permissioned blockchain	Privacy engineering
Sibanda et al, 2025 (60)	Review	NFT healthcare DT	Patient data DT	NFT governance	Compliance-driven
Trivedi et al, 2025 (17)	Survey	RPM with human DT	Wearable DT	Secure RPM blockchain	HIPAA compliance
Wickramasinghe et al, 2025 (61)	Journal	Data-sharing DT	Clinical DT	Secure exchange	Regulatory-aware
Padliya et al, 2025 (8)	Journal	Patient DT prevention	Clinical decision DT	Consent-based blockchain	Ethics & consent
Turab et al, 2023 (35)	Survey	Healthcare DT metaverse	Simulation DT	Secure DT framework	Regulatory discussion
Hao et al, 2025 (16)	Journal	Smart hospital infrastructure	Hospital DT	Access control blockchain	GDPR-aware

Table 2 classifies all included studies according to healthcare field, the range of digital twin, the type of blockchain architecture, and the regulatory orientation. This systematic synthesis allows cross-studying of the patterns of architecture and governance integration of all the included studies.

### 3.3 Blockchain functional distribution across healthcare domains

A mapping table was created to study the patterns of architectural convergence between healthcare domains and blockchain functional roles. The major roles across all the included studies were access control, audit logging, integrity checking, provenance traceability, smart contract, NFT-based ownership, Decentralized Autonomous Organisation (DAO) structures, and federated learning support functions.

**Figure 2:**
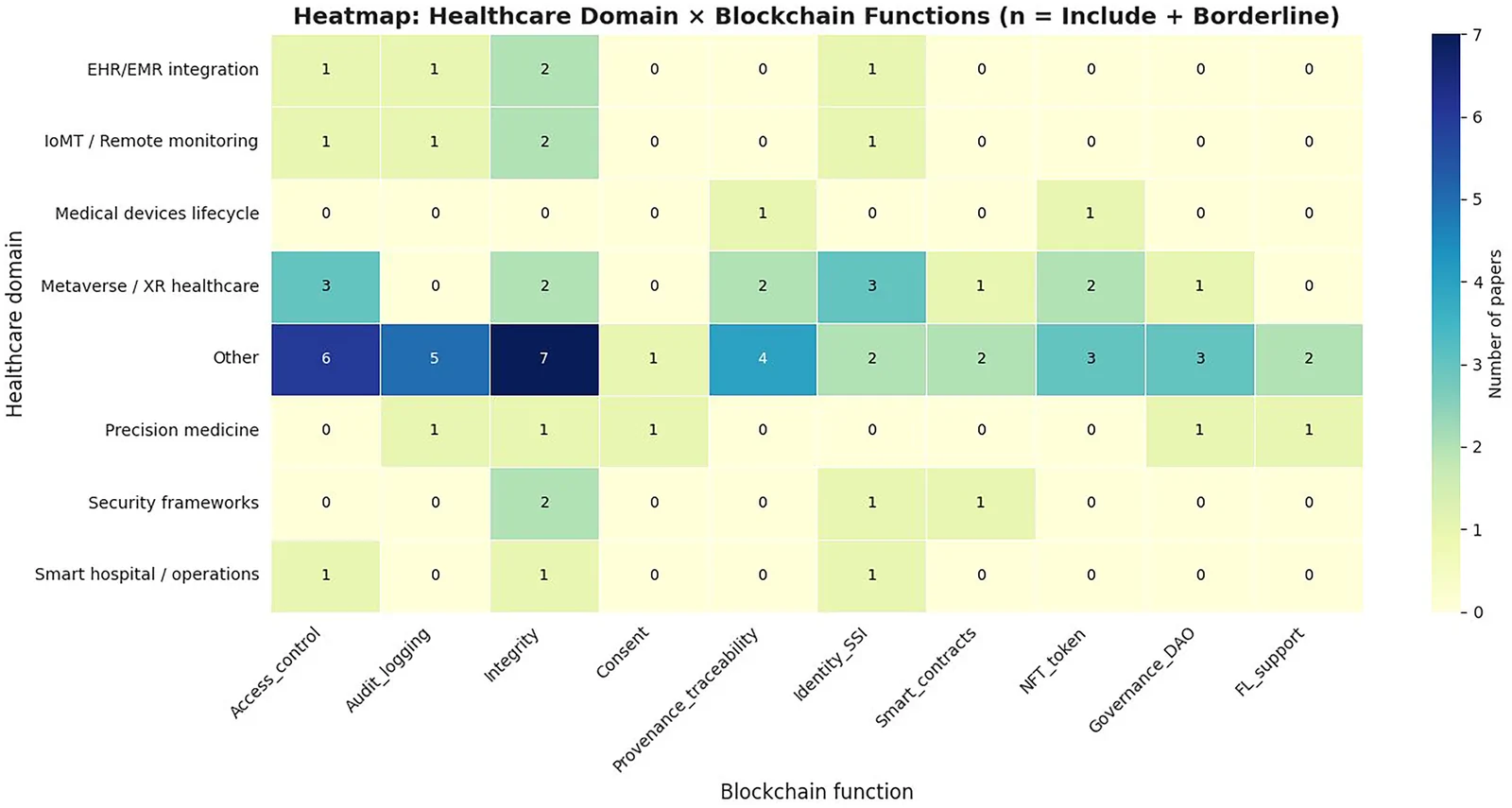
Heatmap of healthcare domains vs. blockchain functional roles (*n* = 55).

Figure 2 illustrates the distribution of roles across different healthcare domains. Furthermore, this heatmap indicates the number of studies that apply each blockchain function in a specific healthcare area. Colour intensity is the number of studies applying a particular blockchain functionality in a particular healthcare field. The visualisation underlines prevailing architectural trends, especially access control and audit logging of patient-level and EHR-integrated systems. This review shows that digital twins on the patient level and EHR-interconnected digital twins are dominated by access control and audit logs (1, 25–27). Metaverse-based and medical device management systems, in turn, seem to be the main systems utilising NFT-based ownership and governance concepts (15, 34, 36).

Moreover, the infrastructure-oriented or cross-domain models constitute a significant share of the literature (53, 54), meaning that most implementations are system-architectural, not tightly integrated into the validation clinical workflow. Such distribution indicates that blockchain-DT integration is still security-focused, with the lack of outcome-oriented clinical deployment works.

### 3.4 Digital twin functional typology

The implementations of digital twins included: patient modelling, predictive simulation, clinical decision support, device-level twins, hospital/system-level twins, behavioural twins, identity-linked twins, synthetic data generation, and educational or training twins.

**Figure 3:**
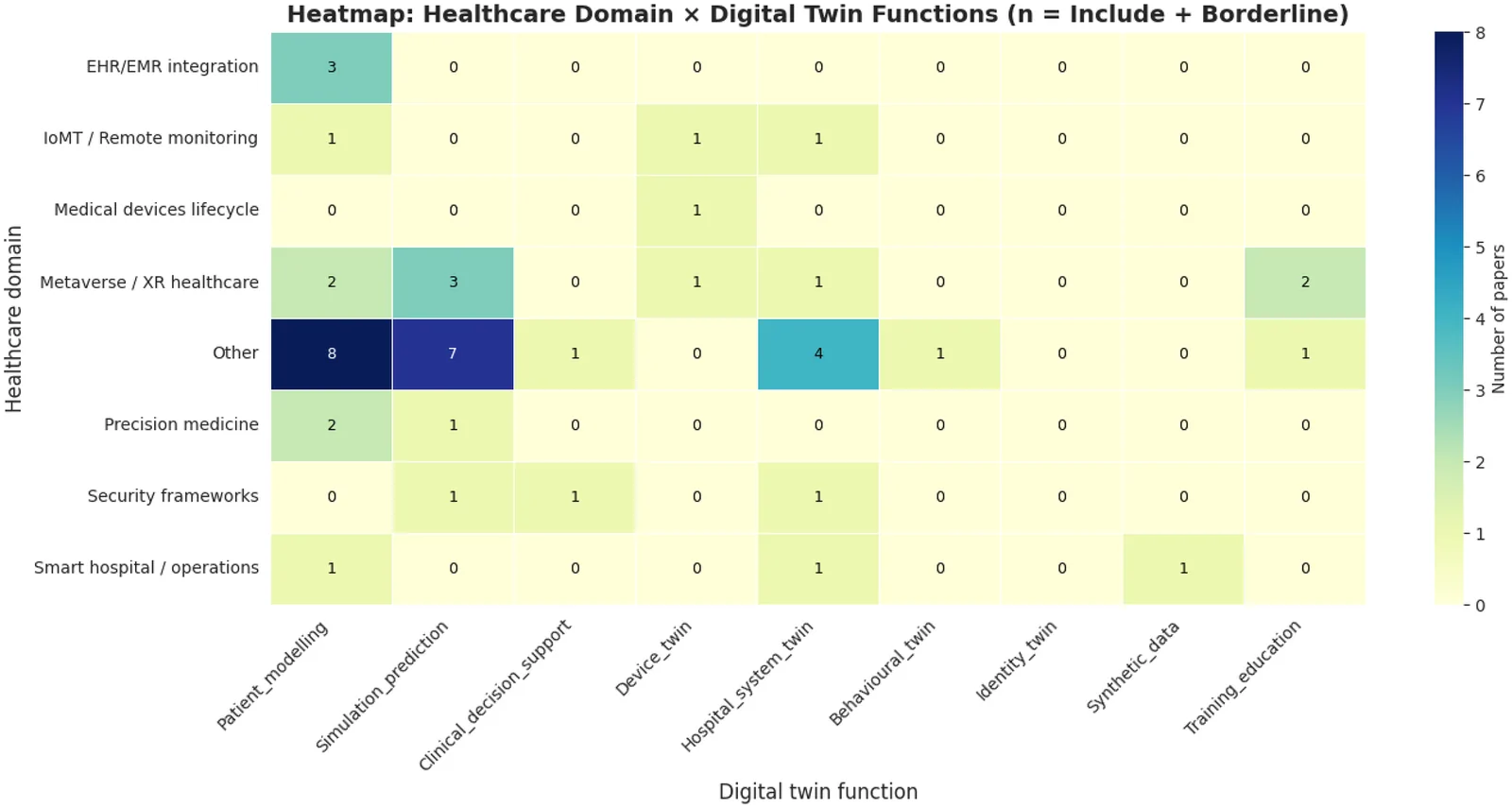
Heatmap of blockchain functional roles across digital twin typologies in healthcare (*n* = 55).

Figure 3 illustrates the connection between the operations of blockchains and digital twins. The matrix in the heatmap (Figure 3) represent the frequency of mapping and co-occurrence of blockchain functional roles and digital twins applied in the work of simulation, system-level twins and device-level architectures, patient modelling. The visualisation shows aggregation of the secure patient modelling systems and predictive simulation systems. Patient modelling and simulation are the most prevalent cases of DT usage, and often they are used along with blockchain-access control and integrity verification (4, 8, 21). Digital twins on the hospital level are found in intelligent hospital designs that focus on secure coordination of infrastructure (7, 16, 51).

Digital twin behaviour and identity linked twins are still relatively limited even though they theoretically coexist with blockchain identity systems. Federated learning assistance can be found mostly in AI-integrated systems (33, 39), meaning that privacy-friendly collaborative learning is not yet developed in practical DT implementations.

Overall, the findings indicate that it focuses on the concept of the static secure data modelling over adaptive and continuously learning digital twin ecosystems.

### 3.5 Ethical and regulatory alignment

Considering the focus on GDPR and HIPAA regulatory frameworks, regulatory alignment was assessed according to explicit references of privacy in the study or a technical safeguard or a direct mapping of the system design to regulatory guidelines.

**Figure 4:**
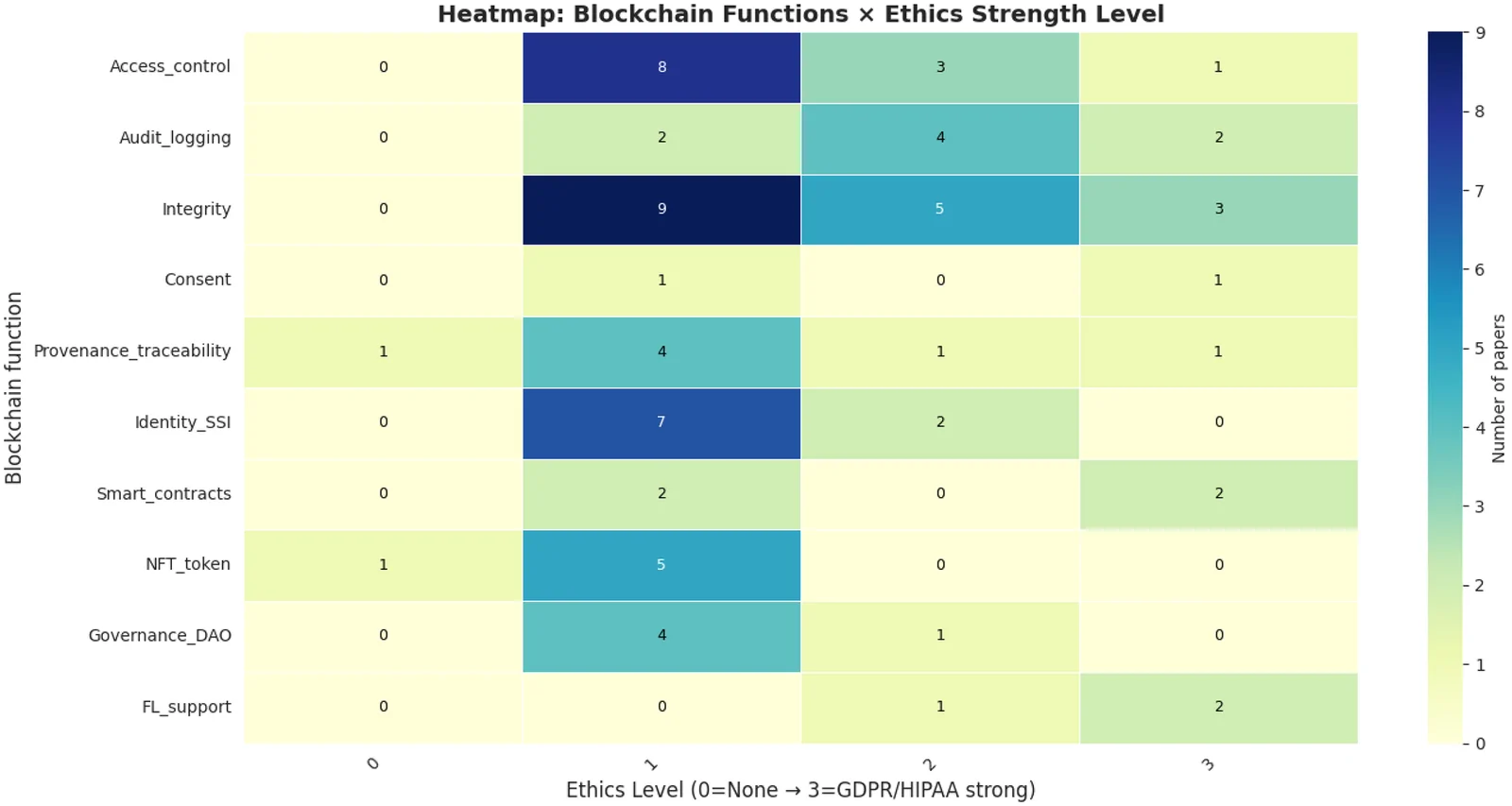
Heatmap of blockchain functions vs. regulatory and ethics strength levels (*n* = 55).

Figure 4 shows the correlation between blockchain functional roles and levels of strength of graded ethics. The roles of blockchain are plotted against the rated alignments with the regulatory aspect, which is 0 (not), 1(mentioned), 2 (technically), and 3 (expressly aligned with the GDPR or HIPAA requirements). However, multi-label classification scheme was used to code blockchain functionalities. Included research articles may fall under more than one category when more than one blockchain mechanism was applied (e.g., smart contracts and tokenization or identity management). Hence, categories are not exclusive to each other. Furthermore, the number emphasises asymmetrical regulation of architectural classification. More often related to explicit regulatory articulation were audit logging, identity management, and consent automation (6, 21, 58). Simultaneously, NFT-based governance frameworks and DAO designs were conceptualised, not tracking the mapping of compliance (36, 60).

To make this classification transparent, the four regulatory alignment levels were defined operationally as follows. Level 0 (not aligned) indicates no reference to GDPR, HIPAA, or an equivalent data protection framework. Level 1 (mentioned) indicates that privacy or regulatory compliance is referenced narratively, without any accompanying technical mechanism. Level 2 (technically aligned) indicates that the study describes a concrete technical mechanism, such as access control, encryption, consent logging, or audit trails, that plausibly supports a regulatory requirement, but without an explicit statement that the mechanism was designed to satisfy a named GDPR or HIPAA provision. Level 3 (expressly aligned) indicates that the study explicitly states that the architecture was designed or evaluated against a named GDPR or HIPAA requirement, or maps specific technical components directly to specific regulatory articles or rules. Two reviewers applied this rubric independently to each study, and disagreements were resolved through discussion, consistent with the coding approach described in the Data Extraction section.

However, multiple studies directly mentioned GDPR or HIPAA compliance (1, 4, 17) but there was a scarcity of cross-jurisdictional compliance modelling. Additionally, the ethical governance discourse was also more common in metaverse-based and AI-driven DT frameworks (32, 47, 54), although with varying degrees of formal regulatory integration.

These data points suggest that the theory of privacy is popular, whereas regulatory engineering of blockchain-based digital twin healthcare is not uniform.

### 3.6 Comparative reporting across publication types

**Figure 5:**
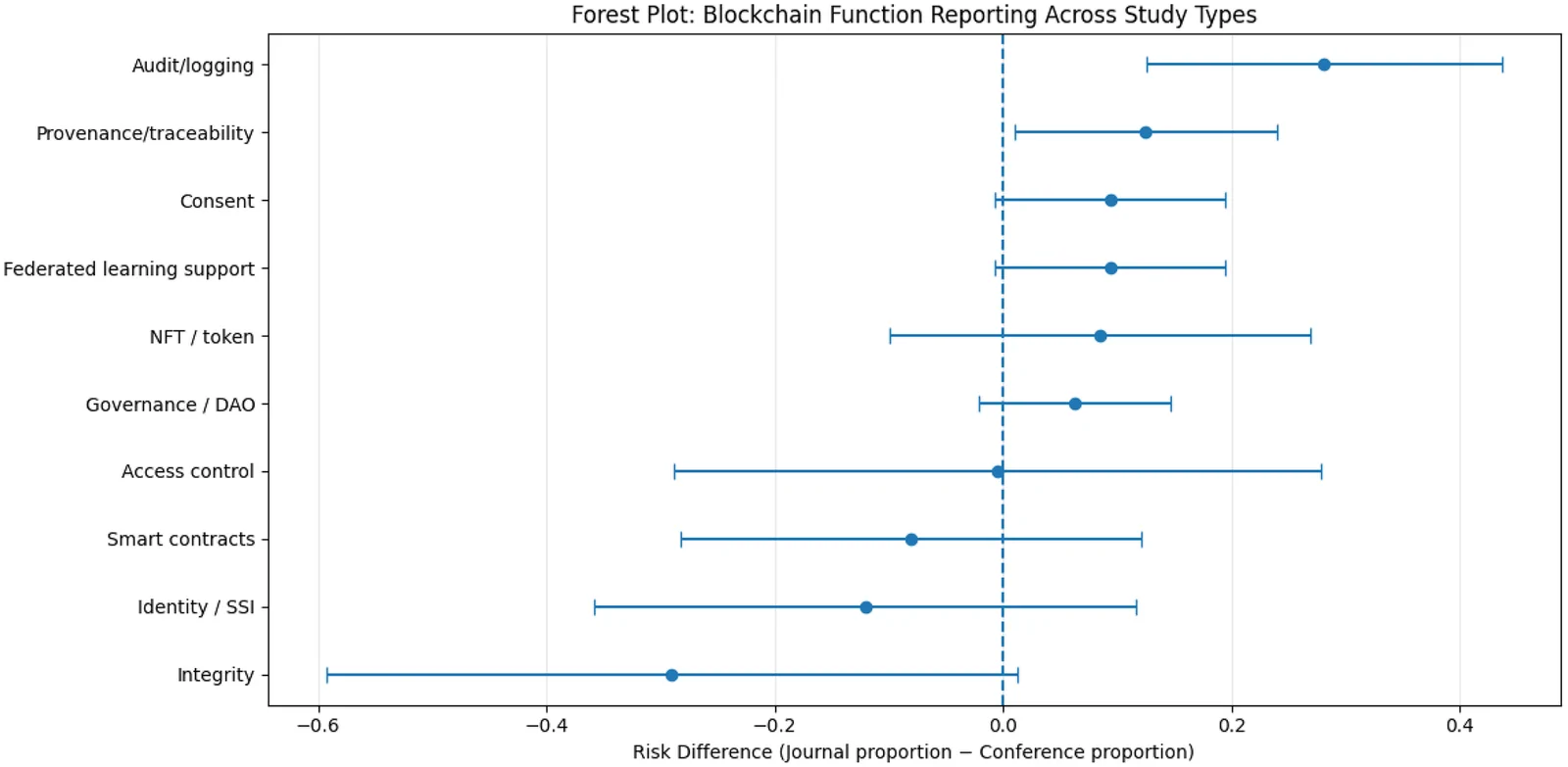
Forest plot of risk differences in blockchain function reporting between journal and conference studies (*n* = 55).

To examine whether publication venue influenced the depth of architectural reporting, risk differences were determined between journal and conference articles on selected blockchain functions (see Figure 5 below).

Moreover, detailed audit logging, governance-layer design, and regulatory challenges were more frequently reported in journal articles (21, 25, 28), and the prototype frameworks and architectural concepts were often presented in the case of conference publications (2, 42, 55). Most functional categories, however, had overlapping confidence intervals, which indicated moderate differences rather than systematic dissimilarities.

The broad gaps of some developed mechanisms of governance indicate small subgroups and demonstrate the insufficient development of several blockchain-DT integration paradigms.

Points and plots in Figure 5 represent risk differences (journal proportion minus conference proportion), with horizontal lines indicating 95% confidence intervals. Positive values indicate higher reporting frequency in journal articles. Overlapping intervals suggest modest variation in reporting depth across publication types.

## 4 Discussion

### 4.1 Clinical and patient-centric digital twin architectures

A large percentage of published works on patient-level digital twins are secured by blockchain-based access control and integrity systems. Early works like El Azzouzi et al. and Akash et al. focused on EHR-based designs, with the addition of blockchain layers to provide immutable logging and safe data transfer (1, 27). In a similar manner, Kumari et al. and Thakur et al. investigated the use of blockchain-supported data audit in the context of patient monitoring systems (12, 14).

Moreover, recent studies have extended this paradigm to predictive and AI-integrated twins. Upadrista et al. and Erol et al. define patient digital twins using blockchain to make predictions and clinical simulations (4, 21). Gao et al. and Ghayvat et al. also incorporate the concept of secure data retrieval and prediction pipelines as part of digital twin ecosystems (28, 51).

Jain et al., and Wang et al. demonstrate the use of blockchain-enabled authentication and integrity verification of distributed sensing infrastructures in the context of IoMT and remote monitoring (31, 41). These implementations enforce dominance in access control and tamper resistance which can be seen in Figures 2, 3.

### 4.2 Metaverse and XR-enabled healthcare twins

The clear thematic group is formed around the healthcare systems enabled by the metaverse. Conceptualisations by Wang et al., Mozumder et al. and Ali et al. view blockchain as a source of trust in XR-oriented digital twins (32, 33, 38). Qayyum et al. and Bhatia et al. build on this, combining NFT based ownership models and smart contract mediated interaction in virtual environments (2, 42).

Hulsen et al. and Naeem et al. discuss wider models of AI-metaverse-healthcare convergence, but regulatory implementation remains limited. Such metaverse-based architectures show more experimentation in terms of governance tokens and distributed trust, but relatively less formal GDPR/HIPAA requirements (46, 54).

### 4.3 Security-centric and infrastructure-level architectures

Several included studies adopt an infrastructure-focused approach. Arastouei et al., Sharma et al. and Bamakan et al. proposed distributed blockchain frameworks that facilitate the healthcare digital twin ecosystems across institutions (34, 37, 52).

Moreover, decentralised biobanking and personalised medicine were proposed in the works of Sanchez et al. and Saratkar et al., in which blockchain maximise the traceability of consent and data provenance (19, 56). These are like the provenance-traceability clusters in Figure 2.

Notably, the decentralised biobanking work of Sanchez et al. was among the few included studies to report a real-world usability and feasibility evaluation rather than a purely conceptual or simulated architecture, illustrating both the feasibility and the current rarity of clinically validated blockchain-digital twin deployments in this literature (56).

Gebreab et al., Okegbile et al., and Shankhdhar et al. also discuss security and cryptographic enhancement, especially in the context of medical device management and integrating digital twins into the IoT (15, 44, 58).

Beyond security architecture, the computational and energy demands of running blockchain consensus alongside continuous, real-time digital twin monitoring have direct implications for clinical feasibility. Permissioned Proof-of-Authority and similar lightweight consensus mechanisms were preferred across most included infrastructure-level studies precisely because they reduce the computational overhead associated with public, Proof-of-Work-style blockchains (34, 37, 52). However, even permissioned designs introduce processing and storage overhead at the point of data capture, and few studies quantified this overhead against the latency and throughput requirements of continuous physiological monitoring or emergency clinical workflows. In settings such as intensive care or ambulatory remote monitoring, where digital twins must ingest and reconcile high-frequency sensor data in near real time, unaddressed consensus latency or energy consumption could constrain clinical adoption regardless of the strength of the underlying security or compliance design. This trade-off between decentralised trust guarantees and real-time performance is therefore a practical, not merely theoretical, barrier to moving these architectures from prototype to bedside use, and is revisited in the Future Research Directions section.

Moreover, quantitative evaluations reported in the literature further support this engineering trade-off. Permissioned Proof-of-Authority blockchains commonly achieve transaction confirmation times in the order of milliseconds to a few seconds with substantially lower computational overhead than Proof-of-Work networks, which may require confirmation times ranging from tens of seconds to several minutes depending on network configuration (11, 64). Likewise, energy consumption reported for PoA deployments is orders of magnitude lower than PoW because consensus relies on trusted validators rather than computational mining (11, 64). Although these benchmarks originate primarily from proof-of-concept evaluations rather than clinical deployments, they reinforce the suitability of permissioned consensus mechanisms for latency-sensitive healthcare digital twin applications (11, 64).

### 4.4 Governance, identity and federated architectures

Digital Twins with identity-centred and federated learning are underdeveloped. Ali et al., Chaddad et al., and Gomaa et al. discuss identity/Self-Sovereign Identity (SSI) architectures, but they lack a detailed implementation (29, 48, 50). Moreover, integration of federated learning along with Digital Twins and blockchain is proposed by Hemdan et al. and Qayyum et al., although the technical implementation of blockchain integration remains underdeveloped (39, 42).

DAO-based governance and tokenised models are only limited within NFT-based applications (9, 36), but decentralised large-scale governance is still merely a theoretical concept that has yet to be empirically tested.

### 4.5 Regulatory alignment and ethical depth

A high level of regulatory consistency such as explicit reference to GDPR/HIPAA is shown by Akash et al., Upadrista et al., Amofa et al., and Erol et al. (1, 4, 6, 21). These papers outline encryption schemes, audit history and privacy preserving computation models.

Simultaneously, few other papers mention privacy in the form of declarations that lack operational alignment to certain regulatory provisions (32, 33, 40).

Moreover, some reviews at infrastructure level were Taleb et al., Ruiu et al., and Mohanraj et al. which recognises the governance issues but do not provide the definition of enforceable compliance architecture (18, 47, 53).

This asymmetry is validated in the ethics heatmap (Figure 4). The mechanisms of integrity and access control are aligned with the formal compliance structures than with smart contracts or consent modules.

Where included studies moved beyond declarative privacy statements, the technical solutions reported to reconcile blockchain immutability with GDPR and HIPAA obligations followed a small number of recurring patterns. The most common was a hybrid on-chain/off-chain design, in which only cryptographic hashes, pointers, or access-control metadata are stored on-chain, while the underlying personal health data is stored off-chain in a conventional database that can be modified or deleted (1, 4, 21). Under this pattern, the GDPR right to erasure is exercised indirectly: the off-chain record is deleted or overwritten, and the corresponding on-chain hash is left as an orphaned, non-identifying reference. A related pattern is cryptographic erasure, in which patient data is encrypted before being written off-chain and the associated decryption key is discarded on request, rendering the data permanently unreadable without altering the ledger itself (17, 21, 58). For HIPAA, the audit-related requirements were most often addressed through blockchain-native audit logging, in which every access, modification, or consent event is written as an immutable transaction, giving covered entities a tamper-evident trail that can support the access-log and audit-control provisions of the HIPAA Security Rule (6, 25, 58). However, few of the included studies described these mechanisms as engineered compliance controls with defined retention periods, key-management procedures, or breach-notification workflows; most were reported as architectural possibilities rather than validated compliance solutions, consistent with the largely conceptual and prototype-stage maturity of the field identified elsewhere in this review.

Interoperability standards received little explicit attention in the reviewed architectures. Although several studies address EHR and EMR integration as a healthcare application area (25, 27, 28), few explicitly discuss alignment with established health-data interoperability standards such as HL7 FHIR, and none of the included studies reported a formal mapping between blockchain-recorded events or digital twin data models and FHIR resources. This is a practical gap, because regulatory-compliant data exchange between blockchain-enabled digital twins and existing hospital information systems is likely to depend on FHIR- or HL7-based interfaces rather than on bespoke data formats. Future architectural work would benefit from positioning blockchain and digital twin components explicitly as FHIR-compliant resource servers or consumers, so that compliance and interoperability can be assessed jointly rather than treated as separate design concerns.

A related limitation is the near-absence of quantitative performance data in the included studies. Although several architectures discuss consensus mechanisms, encryption schemes, and off-chain storage in qualitative terms, very few report measured compliance overhead, transaction latency, or scalability under realistic clinical workloads. Where performance figures were reported, they were typically isolated proof-of-concept benchmarks rather than comparative metrics across architectures, which limits the extent to which this review can quantitatively rank the regulatory or scalability trade-offs of the surveyed designs. Establishing standardised benchmarks for compliance overhead, latency, and throughput is an important methodological gap for future primary studies in this area, and is revisited in the Future Research Directions section.

### 4.6 Ethical implications of blockchain and digital twin convergence in healthcare and clinical data ecosystems

The asymmetry observed between the widespread discussion of privacy principles and the limited operationalisation of regulatory compliance also raises deeper ethical questions about the governance of blockchain-enabled digital twin systems. While many studies describe technical mechanisms such as encryption, access control, and audit logging as ethical safeguards, the literature suggests that these mechanisms primarily address data protection rather than the broader ethical conditions of digital health infrastructures.

Several studies emphasise blockchain as a mechanism for data integrity, traceability, and secure access to digital twin environments (1, 12, 31). These architectures typically rely on immutable ledgers and permissioned access layers to ensure that patient data streams used to construct digital twins remain tamper-resistant and auditable. Similarly, implementations integrating blockchain with predictive digital twins aim to ensure secure data provenance for clinical simulation and personalised medicine applications (3, 4, 28). From a technical standpoint, these architectures represent significant progress in protecting distributed medical data ecosystems.

However, the ethical significance of these mechanisms cannot be reduced to their cryptographic robustness. Healthcare digital twins represent algorithmically mediated representations of patients and clinical systems, built through the continuous aggregation of biological, behavioural, and environmental data streams. As noted by Vasiliu-Feltes et al. and Saratkar et al., these representations introduce new forms of data governance and accountability that extend beyond traditional electronic health record management (13, 19). The ethical challenge therefore concerns not only the protection of data, but also the governance of predictive modelling infrastructures that may influence clinical decisions, treatment planning, and resource allocation.

Another recurring issue in the literature concerns the tension between blockchain immutability and healthcare data rights. Several studies acknowledge potential conflicts between distributed ledger architectures and regulatory requirements such as the right to data modification or deletion under the GDPR (1, 4, 21). Blockchain systems that rely on immutable storage may limit the ability of patients to exercise rights related to data rectification or withdrawal of consent. Although some architectures propose hybrid on-chain/off-chain storage models or cryptographic erasure mechanisms, these solutions remain largely conceptual and rarely tested in real clinical environments.

The literature also reveals that patient agency within blockchain-enabled digital twin systems is often framed primarily through consent management. Smart contracts have been proposed to automate permission structures and data-sharing agreements (6, 8). While such mechanisms can increase transparency in data transactions, they may also shift complex ethical decisions into automated governance layers. In healthcare contexts characterised by asymmetries of knowledge and vulnerability, the automation of consent processes raises questions about whether patients can meaningfully understand or contest the downstream uses of their data within predictive digital twin models.

Emerging architectures exploring metaverse-based healthcare twins further complicate these ethical considerations. Studies describing immersive digital twin environments often emphasise blockchain as a trust infrastructure for identity verification, avatar-based representation, and ownership of digital health data (32, 33, 46). These environments expand the scope of digital health beyond traditional clinical systems into virtual interaction spaces where data governance, identity management, and behavioural modelling intersect. However, regulatory frameworks for these environments remain underdeveloped, and the ethical implications of persistent digital patient representations in immersive environments remain largely unexplored.

Another dimension concerns distributive justice and technological maturity. The review demonstrates that many blockchain–digital twin systems remain at the conceptual or prototype stage, particularly in conference publications (2, 36, 44). As a result, many proposed architectures rely on idealised assumptions regarding interoperability, institutional coordination, and data availability. Without empirical validation in diverse healthcare settings, these systems risk reinforcing existing inequalities in digital health infrastructure, particularly between highly resourced institutions and healthcare environments with limited technological capacity.

Similarly, decentralised governance models such as NFT-based data ownership or DAO-based health data ecosystems have been proposed in several studies (15, 34, 60). While these models aim to increase patient control over health data, they also introduce new governance challenges regarding responsibility, liability, and regulatory oversight. Healthcare systems require accountability structures capable of addressing clinical risk and institutional obligations, which may be difficult to reconcile with fully decentralised governance mechanisms.

Taken together, the literature suggests that blockchain-enabled digital twin systems are currently being developed primarily as secure technical infrastructures rather than fully articulated ethical governance systems. The dominant architectural focus on integrity verification, access control, and secure data exchange demonstrates that trust in these systems is largely conceptualised in technical terms. However, ethically robust healthcare infrastructures require more than cryptographic assurance. They also require governance mechanisms that ensure transparency, accountability, contestability, and equitable participation across healthcare stakeholders.

Existing standardisation efforts outside the blockchain and digital twin literature may offer a useful starting point for operationalising this governance layer. For example, IEEE P7000, the IEEE standard model process for addressing ethical concerns during system design (63), provides a structured process for identifying stakeholder values and translating them into verifiable design requirements, and could be adapted to guide the ethical governance of blockchain-enabled digital twin architectures in a way that complements, rather than duplicates, GDPR- and HIPAA-oriented technical compliance work. None of the included studies referenced such process-oriented ethics standards, suggesting an opportunity for closer engagement between the blockchain-digital twin engineering literature and established ethics-by-design frameworks.

Furthermore, one practical reason may be that IEEE P7000 specifies a process for embedding ethical values into engineering design rather than prescribing concrete software components or implementation checklists (63). Consequently, blockchain and digital twin development teams may find it easier to implement measurable technical controls such as encryption, access logging, or consent management than to operationalise value-driven stakeholder analysis throughout the software development lifecycle (11, 63). Developing implementation guidance that translates IEEE P7000 principles into reusable blockchain architecture patterns and software engineering workflows therefore represents an important opportunity for future research (63).

Consequently, future research on blockchain-based digital twin systems should move beyond the language of regulatory awareness and address the integration of ethical governance directly into system architecture. This includes the development of dynamic consent frameworks, transparent audit mechanisms interpretable by clinicians and patients, mechanisms for correcting or updating patient representations within digital twin models, and governance frameworks capable of balancing decentralisation with institutional responsibility. Such developments will be essential if blockchain-enabled digital twins are to evolve from experimental infrastructures into clinically legitimate and ethically sustainable components of digital health ecosystems.

### 4.7 Comparative maturity across publication types

Works presented in conferences are more likely to focus on proof-of-concept architectural designs (2, 36, 44). However, journal articles like Gao et al., Upadrista et al., and Amofa et al. have more detailed reports of privacy mechanisms and regulatory implementation (4, 6, 28).

The forest plot (Figure 5) proves that even though the depth of reporting is different, the emphasis level on the functional areas is the same across all domains.

## 5 Strengths and limitations

To the best of our knowledge, this review is the first systematic scoping literature review of blockchain-based digital twin platforms in healthcare that are explicitly analysed through a combined architectural and functional regulative domains along with a quality assessment. Furthermore, this review also managed to provide the methodological transparency and reproducibility by combining PRISMA-based systematic screening with structured data extraction and MMAT quality appraisal (Supplementary File 3). Moreover, multidimensional co-occurrence heatmaps provided the opportunity to systematically map healthcare domain, blockchain functionality, different types of digital twin integrations and levels of regulatory challenges among all 55 studies included. This is a more consistent methodology because it is not a strictly narrative review but allows architectural comparison.

However, there are several limitations in the study. Firstly, even with the thorough coverage of 3 major databases (Scopus, PubMed, Web of science) there were no emergent preprints or grey literature included, possibly leaving out the initial technical developments. Secondly, an interpretive judgement was needed to divide them into healthcare domains and the level of ethics strength, but predetermined coding criteria was used to achieve uniformity. Thirdly, the growing development of blockchain and digital twin environments can make some technical architectures obsolete due to the development of interoperability standards and regulatory guidance. Lastly, the regulatory strength was rated, but real compliance verification was not done separately as it was evaluated based on author-reported compliance. Fifthly, the geographic distribution of the included evidence base was skewed towards Europe and Asia, with comparatively few empirically grounded, HIPAA-focused implementations originating from the United States; this imbalance should be considered when generalising the regulatory findings to US healthcare settings. Sixthly, the evidence base is dominated by conceptual and prototype-stage architectures, with very few studies reporting pilot or real-world clinical deployments, which limits conclusions about the operational feasibility of the reviewed designs. Finally, while ethical frameworks and regulatory principles were widely discussed, practical enforcement mechanisms, such as automated smart-contract compliance audits, were rarely reported, so this review characterises the maturity of ethical discourse rather than the maturity of ethical enforcement.

Irrespective of these limitations, the review offers a solid structural mapping of a fast-growing interdisciplinary field.

## 6 Future research directions

The results of this systematic scoping review indicate that blockchain-based digital twin systems in the domain of healthcare are no longer at the stage of theoretical architectural designs but the practical clinical systems. However, considerable research and implementation issues must be overcome to enable large-scale implementation in clinical practice. The future research should focus on the next step of developing framework-based demonstrations to clinical and regulation-compliant deployments.

Moreover, one of the key directions is related to clinical validation and longitudinal implementation of healthcare digital twins. Although several studies suggest the use of patient-specific modelling and simulation environments, their empirical validation in the context of hospital workflows has not been done (4, 6, 25). The future research should focus prospective clinical researches to establish the predictive power, capacity to optimise therapy, and trustworthiness of digital twins in forecasting chronic disease management, surgical planning, and personalised medicine environments (47, 55).

In addition, scalable governance and decentralised identity management represent an important research direction that remains essential. New self-sovereign identity (SSI) and blockchain-based governance systems have a high potential to facilitate patient-controlled healthcare systems (18, 56). However, some forms of governance, including decentralised autonomous organisations (DAOs), are very hypothetical in the healthcare sector (34). The future work should hence explore the hybrid governance constructs that can be used to balance the principles of decentralisation with those of institutional accountability, clinical regulation as well as ethical oversight demands.

Another key area is regulatory integration. Even though GDPR and HIPAA aware architectures are increasingly in discussion, technical implementations hardly support dynamic consent management or auditability or enforcement of cross-border compliance (1, 28). Furthermore, the next-generation systems will need to incorporate compliance features into blockchain based smart contracts so that they can provide automated policy enforcement and regulatory audit of distributed healthcare systems (58).

**Figure 6:**
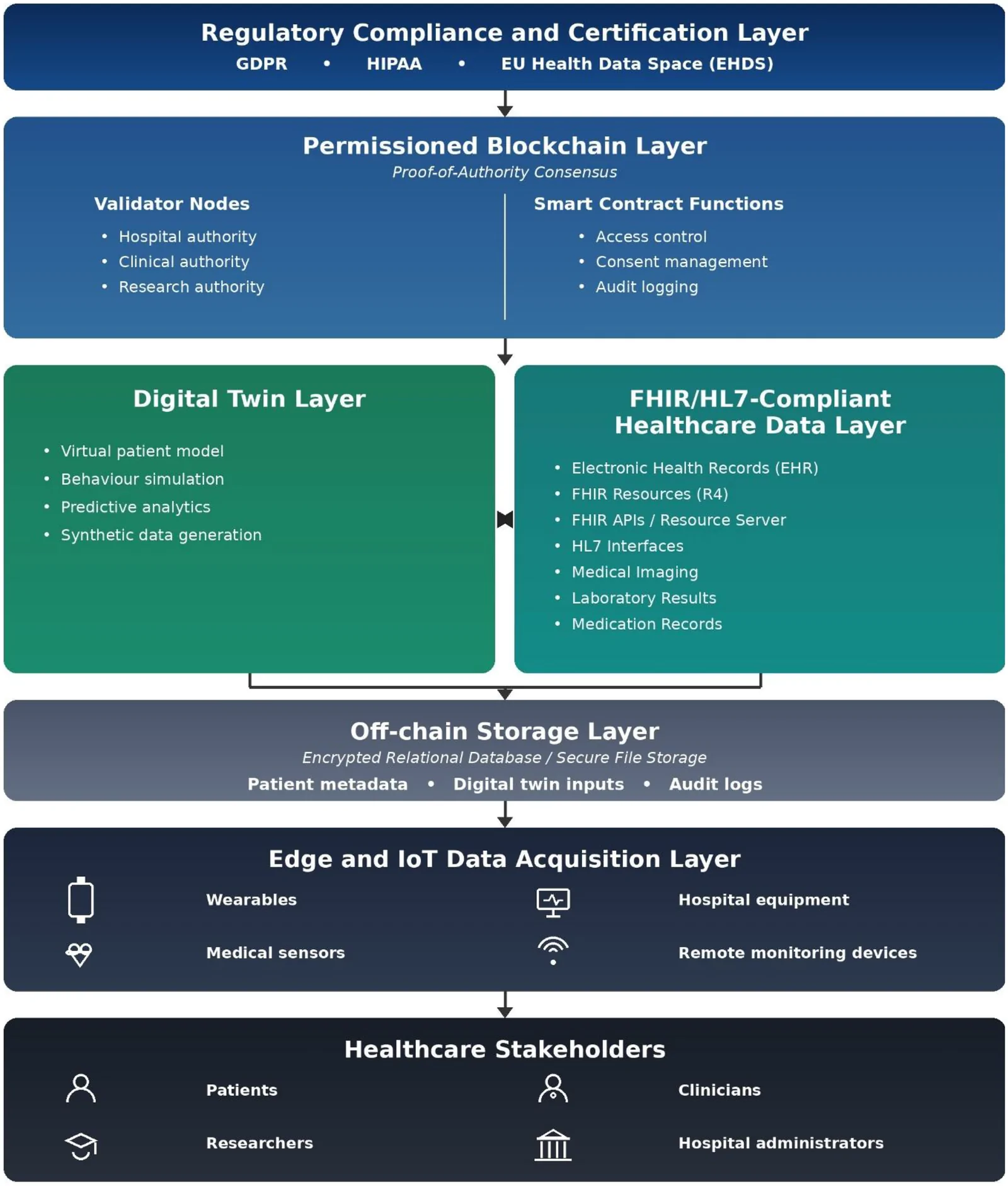
Proposed reference architecture for blockchain-enabled digital twin systems in healthcare with FHIR/HL7-compliant interoperability. The Healthcare Data Layer intentionally exposes FHIR-compliant interfaces to enable interoperability with existing EHR systems while preserving blockchain-based governance and auditability.

Unlike the architectures identified in the reviewed literature, the proposed reference architecture in Figure 6 explicitly incorporates a FHIR/HL7-compliant Healthcare Data Layer to enable standards-based interoperability between blockchain-enabled digital twins and existing hospital information systems.

Future work could discuss and focus on integrated architectures that can ensure a combination of digital twin modelling, blockchain governance, and regulatory compliance mechanisms in one healthcare data ecosystem. This type of architecture may incorporate wearable, hospital equipment, medical sensors, and serious gaming applications streams of patient data into a layered structure where the digital twin models and healthcare records are synchronised by secure off-chain databases and governance and verification by a permissioned Proof-of-Authority blockchain, as shown in Figure 6 (4, 11, 33, 62). This model has smart contracts that govern access control, consent management and audit logging, and regulatory certification layers that consist of GDPR, HIPAA and European Health data space compliance and trust among the involved clinical institutions. This form of architecture builds on the earlier studies of blockchain-based healthcare data governance and data monitoring systems, showing how digital twins, IoT data collection, and regulatory-compliant blockchain systems can be incorporated into one framework of future intelligent healthcare systems (1).

Moreover, the combination of federated learning and artificial intelligence to blockchain-secured digital twin ecosystems is likely to characterise the next generation of smart healthcare platforms. The federated analytics tool allows institutions to learn together without using sensitive patient information, which would solve privacy and interoperability limitations at the same time (3, 33). The development of adaptive and continuously learning digital twins capable of updating the patient models in real time is a particularly promising research direction.

Lastly, the research in this domain needs to be expanded in the future to address sustainability and computational power concerns regarding the implementation of blockchain in digital twins in healthcare settings. In addition, lightweight distributed architectures, consensus optimisation, and energy-efficient trust mechanisms are also required to make healthcare systems feasible for large-scale implementation (7, 53).

Finally, the development of these directions will play a critical role in transforming blockchain-based digital twins as experimental infrastructures into reliable clinical decision ecosystems.

## 7 Conclusion

This systematic review represents an analysis of the existing literature on blockchain-based digital twins in the healthcare sector that combines technical, ethical, and governance aspects in all the 55 articles included in the review. Furthermore, the facts reveal that there is a convergence of digital twin modelling and blockchain-based trust infrastructures rapidly towards the long-standing problem associated with interoperability of healthcare, data integrity, privacy protection, and decentralised governance.

In addition, this review constantly discusses blockchain technologies as the trust layer that ensures safe data exchange, identity management, auditability, and provenance tracing, whereas digital twins make it possible to simulate patients, provide predictive analytics, and optimise processes. This joint architecture lays a paradigm shift on continuously changing virtual representation of patients, medical devices and health care system that can support precision medicine and intelligent clinical decision-making (25, 27, 47).

Although there has been significant progress to be achieved, the review states that the current implementations are mostly in conceptual or architectural phases. Furthermore, there are still not many fully operational clinical deployments that combine regulatory compliance and governance transparency and scalable interoperability. Moreover, the concept of ethical and privacy is generally accepted but not integrated into the system design, which suggests that there is always a misalignment between regulations and technology implementation (1, 13, 28).

However, the ethical implications of blockchain-enabled digital twins extend beyond regulatory compliance and data protection alone. As the literature reviewed suggests, these systems increasingly function as infrastructures for modelling, interpreting, and governing patient data ecosystems. In this sense, the ethical challenge is not limited to securing health data but also concerns how digital twin representations influence clinical decision-making, patient agency, and accountability within distributed healthcare networks. Embedding ethical governance directly into system architecture through transparent consent mechanisms, accountable data governance, and interpretable audit structures will therefore be critical for ensuring that these technologies remain aligned with the values and responsibilities of clinical practice.

Notably, this systematic scoping review underlines the idea that decentralised privacy-preserving digital infrastructures that incorporate blockchain as a means of trusted collaboration and digital twins as predictive and personalised healthcare delivery will probably form the basis of future healthcare ecosystems. Furthermore, blockchain-enabled digital twins can additionally be considered part of the future of digital health systems due to their integration with federated intelligence and immersive clinical environments, along with real-time patient monitoring (3, 42, 58).

Beyond synthesising the literature, this review makes three principal contributions. First, it proposes a compliance-oriented taxonomy that categorises blockchain-enabled healthcare digital twins according to functional architecture, regulatory maturity, and ethical governance rather than application domain alone. Second, it synthesises the engineering trade-offs between lightweight permissioned consensus mechanisms and practical deployment requirements, highlighting the balance between decentralised trust and clinical performance. Third, it extends existing architectural discussions by positioning process-oriented ethics frameworks, including IEEE P7000, alongside GDPR and HIPAA as complementary foundations for trustworthy healthcare digital twin engineering.

Finally, digital twin enabled by blockchain has tremendous transformational opportunities in healthcare, but to reach clinical readiness, coordinated efforts must be made in the domains of validation, governance engineering, regulatory integration, and scalable system design. The interdisciplinary cooperation between engineers, clinicians, policymakers and regulatory authorities will continue to ensure that the realisation of trustful, ethically consistent and globally interoperable digital healthcare infrastructures is achieved.

## Data Availability

The original contributions presented in the study are included in the article/Supplementary Material, further inquiries can be directed to the corresponding author/s.
