# Supplementary material for: Blockchain-enabled digital twin systems in healthcare: a systematic scoping review of architectural integration, privacy, governance, ethical challenges, and regulatory solutions under GDPR and HIPAA
Source: Front Digit Health. 2026 Aug 12;8:1898383. doi: 10.3389/fdgth.2026.1898383 (PMC13539559; doi:10.3389/fdgth.2026.1898383)
Supplement: Supplementary file 2 [file Supplementaryfile2.docx]

**Search String:**

**Scopus:**

*TITLE-ABS-KEY ( ( "blockchain" OR "block-chain" OR "smart contract*" OR "distributed ledger" ) AND ( "digital twin" OR "digital twins" OR "virtual patient" ) AND ( health OR healthcare OR "health care" OR medical OR hospital OR clinical ) AND ( ethics OR "ethical consideration*" OR privacy OR "data protection" OR HIPAA OR GDPR ) )*

**PubMed:**

*(	("blockchain"[Title/Abstract] OR "block-chain"[Title/Abstract] OR "smart contract"[Title/Abstract] OR "distributed ledger"[Title/Abstract])	AND	("digital twin"[Title/Abstract] OR "digital twins"[Title/Abstract] OR "virtual patient"[Title/Abstract])	AND	("health"[Title/Abstract] OR "healthcare"[Title/Abstract] OR "health care"[Title/Abstract] OR "medical"[Title/Abstract] OR "hospital"[Title/Abstract] OR "clinical"[Title/Abstract])	AND	("ethics"[Title/Abstract] OR "ethical consideration"[Title/Abstract] OR "privacy"[Title/Abstract] OR "data protection"[Title/Abstract] OR "HIPAA"[Title/Abstract] OR "GDPR"[Title/Abstract]) )*

**Web of Science:**

*TS = (	("blockchain" OR "block-chain" OR "smart contract*" OR "distributed ledger")	AND	("digital twin" OR "digital twins" OR "virtual patient")	AND	(health OR healthcare OR "health care" OR medical OR hospital OR clinical)	AND	(ethics OR "ethical consideration*" OR privacy OR "data protection" OR HIPAA OR GDPR) )*
